# An Adaptive Altitude Information Fusion Method for Autonomous Landing Processes of Small Unmanned Aerial Rotorcraft

**DOI:** 10.3390/s121013212

**Published:** 2012-09-27

**Authors:** Xusheng Lei, Jingjing Li

**Affiliations:** Science and Technology on Inertial Laboratory, Beijing 100191, China; E-Mail: 001rose001@sina.com

**Keywords:** small unmanned aerial rotorcraft, wavelet filter, altitude information fusion, adaptive extended Kalman filter

## Abstract

This paper presents an adaptive information fusion method to improve the accuracy and reliability of the altitude measurement information for small unmanned aerial rotorcraft during the landing process. Focusing on the low measurement performance of sensors mounted on small unmanned aerial rotorcraft, a wavelet filter is applied as a pre-filter to attenuate the high frequency noises in the sensor output. Furthermore, to improve altitude information, an adaptive extended Kalman filter based on a maximum *a posteriori* criterion is proposed to estimate measurement noise covariance matrix in real time. Finally, the effectiveness of the proposed method is proved by static tests, hovering flight and autonomous landing flight tests.

## 1. Introduction

With the ability to land vertically, small unmanned aerial rotorcraft (SUAR) have an irreplaceable role in civil applications [1]. Thus, they have been widely used in many areas, including road traffic monitoring, city building surveillance and power line inspection, *etc.* [2,3].

SUAR is a complex multi-input and multi-output (MIMO) system. Compared with the hovering and straight flight processes, there exists land disturbance in the landing process [4]. High performance altitude information is the basis factor for SUARs to realize stable landing control [5]. Due to the constraints of weight and size, sensors with low size and low performance are often used by SUARs, including micro-electronic mechanic system (MEMS) accelerometers and gyroscopes, barometers, the global positioning system (GPS) and ultrasonic sensors.

Integrated by the Euler equations, or quaternions, SUAR can get the corresponding aircraft attitude angles and position information, however, inertial sensors, especially gyroscopes, have fixed bias, drift bias, asymmetric scale factor errors and temperature-varying biases, causing the integration results to drift from true attitude [6]. GPS can provide absolute position and velocity information [7], but GPS information is easily affected by sources of interference [8]. Furthermore, GPS has low data frequency to get position and velocity information for a SUAR system [9]. Based on the relationship between the air pressure and the altitude, barometers can provide altitude information [10], but they are easily affected by wind disturbances, air fluctuations, and temperature [11]. Ultrasonic sensors are also often used in SUAR systems. Although they can provide high performance altitude measurements, they have measurement region limitations. When the altitude of SUAR surpasses the upper bound of ultrasound, the measurement results will have errors, therefore all the current sensors have limitation for SUAR to realize stable landing control.

Using filter methods, system can get high performance information based on different sensors. The most used filtering method is the extended Kalman filter (EKF) [12]. With the predict and update theory, Beard has used EKF to realize attitude acquisition for a unmanned aerial vehicle [13]. Nevertheless, poor performance or even divergence arising from the linearization implicit in EKF has led to the development of other filters [14]. The unscented Kalman filter (UKF) is also used in UAV systems [15]. Based on second or higher-order approximations of nonlinear functions, UKF can estimate the mean and covariance of state vectors [16]. With UKF, Seung realized target relative position and velocity determinations for follower UAV systems [17]. However, UKF is sensitive to the statistical distribution of the stochastic processes [18]. Based on the concept of sequential sampling and Bayesian theory, particle filtering (PF) is also used in dealing with nonlinear and non-Gaussian noise in SUAR systems. Kamrani used PF for efficient path planning of a UAV [19], but its computational demands are too complex. Wavelet analysis has also been widely used for its time and frequency domain convenience and it can effectively eliminate high frequency noise. Tsiotras used a wavelet transform to construct an approximation of the environment at different levels for small UAVs [20].

Inspired by the discussion above, an adaptive extended Kalman filter (AEKF) method based on the wavelet filter is proposed to get high performance altitude information for a SUAR during the autonomous landing process. The wavelet decomposition and reconstruction method is used to restrain the high frequency noise in the barometer, ultrasonic and GPS sensor information. Since the measurement noise is greatly changed after wavelet filtering, an AEKF based on a maximum *a posteriori* criterion is proposed to estimate the measurement noise matrix in real time to get high performance altitude information.

The paper is organized as follows: the dynamic model of the SUAR system is described in Section 2. The wavelet decomposition and reconstruction method is presented in Section 3. In Section 4, an AEKF based on a maximum *a posteriori* criterion is proposed to improve altitude information. The simulation and test results confirm the effectiveness of the proposed method in Section 5. Finally, conclusions are drawn in Section 6.

## 2. The Dynamic Model of SUAR

### 2.1. The State Model

For SUAR, altitude information is mainly controlled by the main rotor speed and longitudinal cyclic input. Therefore, the simple altitude dynamic model for SUAR can be defined as:

(1) $$\{\begin{array}{c} {\dot{x}}_{1}=f_{1}=x_{2}\\ {\dot{x}}_{2}=f_{2}=a_{0}+a_{1}x_{2}+a_{2}x_{2}^{2}+(a_{3}+a_{4}x_{4}-\sqrt{a_{5}+a_{6}x_{4}})x_{3}^{2}\\ {\dot{x}}_{3}=f_{3}+u_{1}=a_{7}+a_{8}x_{3}+(a_{9}\sin x_{4}+a_{10})x_{3}^{2}+u_{1}\\ {\dot{x}}_{4}=f_{4}=x_{5}\\ {\dot{x}}_{5}=f_{5}+u_{2}=a_{11}+a_{12}x_{4}+a_{13}x_{3}^{2}\;\sin x_{4}+a_{14}x_{5}+u_{2} \end{array}$$

where *x_i_* = (*h*, *ḣ*, *w*, *θ*, *θ̇*)*^T^*, *i* = 1,⋯,5. The term *h* is the estimated altitude measured by barometer, GPS, and ultrasonic sensors, *w* is rotor speed of main rotor, *θ* is the longitudinal angle of rotor blade speed, *u* = (*u*_1_, *u*_2_)*^T^* is the corresponding input, *u*_1_ and *u*_2_ are throttle and collective input respectively, playing an important role in longitudinal cyclic input, lateral cyclic input and blade speed. *a_j_*(*j* = 1,2,⋯,15) are unknown identification parameters, obtained by the adaptive genetic method [21]. Therefore, the altitude state model of SUAR can be defined as follows:

(2) $$\dot{h}=f(h,w,\theta ,\dot{\theta })$$

### 2.2. The Measurement Model

The output accuracy of a barometer is mainly affected by the high frequency noise and constant error which is related to air pressure and temperature. The high frequency noise can be restrained largely by a wavelet filter. Thus, the barometer output *h_b_* mainly includes a constant error *ε_b_* and measurement noise *v*_1_. The function of the *h_b_* can be defined as follows:

(3) $$h_{b}=h+{ɛ}_{b}+v_{1}$$

where *h* is the altitude of the SUAR system.

With the location method of the ranging interchange theory, DGPS can provide position information for SUAR systems with sub-meter performance. The output of DGPS can be defined as follows:

(4) $$h_{g}=h+v_{2}$$

where *h_g_* is the output of DGPS, and *v*_2_ is measurement noise

Ultrasonic sensors can provide high performance altitude information from 0.15 m to 6.05 m, and the error is less than 1 millimeter. When the altitude surpasses the upper limitation, the output of ultrasonic sensor fluctuates greatly. Therefore, the output of the ultrasonic sensor can be defined as follows:

(5) $$h_{u}=\{\begin{array}{ll} w_{lu} & h\geq 6.05m\\ w_{u} & 0\leq h<6.05 \end{array}$$

where *h_u_* is the output of ultrasonic sensor, *w_u_* is the random error within the bounds and *w_lu_* is random error without bounds.

When a SUAR finishes a certain task at low altitude, there exists land disturbance causing an increase of barometer error. Ultrasonic sensors can provide high precision altitude information for SUARs at low altitude, therefore the measurement matrix can be constructed with inputs from different sensors.

#### Case 1

If the integrated navigation altitude is larger than 6 m, the SUAR is beyond the range of the ultrasonic sensor. The output of barometer and DGPS are fused. Thus, the constant error of barometer sensor can be revised by the DGPS. The measurement equation can be defined as:

(6) $$\left[\begin{array}{c} h_{b}\\ h_{g} \end{array}\right]=\left[\begin{array}{ll} 1 & 1\\ 1 & 0 \end{array}\right]\;\left[\begin{array}{c} h\\ {ɛ}_{b} \end{array}\right]+\left[\begin{array}{c} v_{1}\\ v_{2} \end{array}\right]$$

#### Case 2

If the integrated navigation altitude is less than 6 m, the barometer is easily affected by land disturbance. The output of DGPS and ultrasonic sensor are used to construct the measurement vector. The measurement equation can be defined as follows:

(7) $$\left[\begin{array}{c} h_{g}\\ h_{u} \end{array}\right]=h+\left[\begin{array}{c} v_{2}\\ w_{u} \end{array}\right]$$

**Figure 1.**
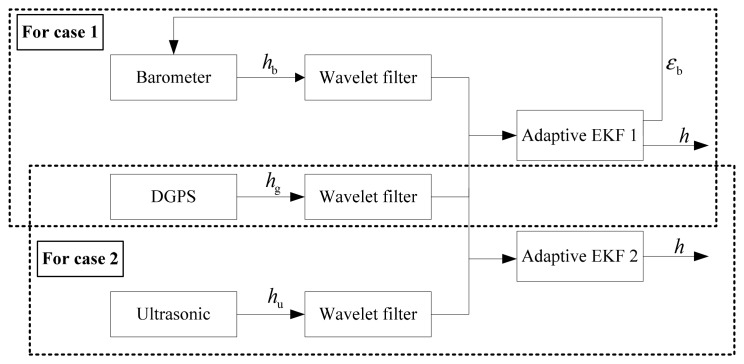
The scheme of altitude fusion.

Therefore, the measurement equation can be expressed as:

(8) $$Z_{k}=H_{k}X_{k}+V_{k}$$

where *H_k_* is the state vector, *Z_k_* is the measurement vector, *H_k_* is the measurement matrix and *V_k_* is the measurement noise vector. With the wavelet filter, the high frequency noises can be largely eliminated. Then, using the AEKF based on maximum *a posteriori* criterion, the altitude *h* and the constant error *ε_b_* can be estimated unbiasedly. The whole procedure is shown in Figure 1.

## 3. The Wavelet Decomposition and Reconstruction Method

To get high precision altitude information, it is necessary to use a data filter to deal with high frequency noises in the output of barometer sensor, DGPS, and ultrasonic sensor. Wavelet analysis is a time and frequency domain method, having good representation for partial signal characteristics, therefore, a wavelet filter is used here as a tool to reduce high frequency noises in the sensor information. Lifting-based wavelet transform implementation has shown high potential in reducing the number of computations, so it is used to reduce computation burden in real tasks. It includes three steps:

1. Split: splitting the original signal *s^j^* = {*s^j^,k*|0 ≤ *k* < 2*^j^*} into even and odd ones. That is:
  (9) $$\{\begin{array}{l} \text{Split}(s^{j})=(E_{j-1},O_{j-1})\\ E_{j-1}=\{E_{j-1,k}=s_{j,2k}\},O_{j-1}=\{O_{j-1,k}=s_{j,2k+1}\} \end{array}$$
2. Predict: defining the detailed representation characteristics by choosing a predictor:
  (10) $$O_{j-1}-=P(E_{j-1})$$
3. Update: averaging the signal of rough representation against original signal:
  (11) $$E_{j-1}+=U(O_{j-1})$$

The basic principle of lifting scheme is to factorize the polyphase matrix of a wavelet filter into a sequence of alternating upper, lower triangular matrices and a diagonal matrix with constants. The factorization is obtained by using an extension of the Euclidean algorithm. The resulting formulation can be implemented by means of banded matrix multiplications.

Suppose that the z-transform of wavelet filter *h* = {*h_k_*, *k* ∈ *Z*} can be defined as *h*(*z*). Let *ĥ*(*z*) and *ĝ*(*z*) be the low and high pass analysis filters, and *h*(*z*), *g*(*z*) be the low and high pass synthesis filters. *ĥ*(*z*), *ĝ*(*z*), *h*(*z*) and *g*(*z*) are biorthogonal filters. The filters can be divided into even and odd parts as:

(12) $$\{\begin{array}{l} \hat{h}(z)={\hat{g}}_{e}(z^{2})+z^{-1}{\hat{g}}_{o}(z^{2})\\ \hat{g}(z)={\hat{g}}_{e}(z^{2})+z^{-1}{\hat{g}}_{o}(z^{2})\\ h(z)=h_{e}(z^{2})+z^{-1}h_{o}(z^{2})\\ g(z)=g_{e}(z^{2})+z^{-1}g_{o}(z^{2}) \end{array}$$

The polyphase matrices are then defined as:

(13) $$\{\begin{array}{c} \hat{P}(z)=\left[\begin{array}{ll} {\hat{h}}_{e}(z) & {\hat{h}}_{o}(z)\\ {\hat{g}}_{e}(z) & {\hat{g}}_{o}(z) \end{array}\right]\\ P(z)=\left[\begin{array}{ll} h_{e}(z) & h_{o}(z)\\ g_{e}(z) & g_{o}(z) \end{array}\right] \end{array}$$

If the (*ĥ*, *ĝ*) is a complementary filter pair, then *P̂*(*z*) can be factored as follows:

(14) $$P(z)=\prod_{i=m}^{m}\;\left[\begin{array}{cc} 1 & s_{i}(z)\\ 0 & 1 \end{array}\right]\;\left[\begin{array}{cc} 1 & 0\\ t_{i}(z) & 1 \end{array}\right]\;\left[\begin{array}{cc} K & 0\\ 0 & K^{-1} \end{array}\right]$$

where *K* is a constant value.

**Figure 2.**
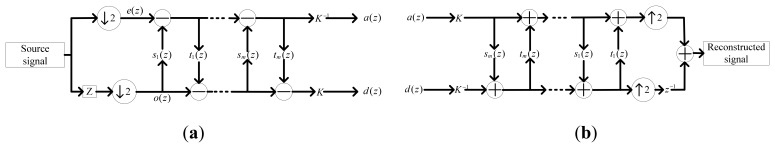
The polyphase-based wavelet transform in lifting scheme. (**a**) The wavelet analysis process. (**b**) The wavelet reconstruction process.

Therefore, the low pass samples are multiplied by the time domain equivalent of *s_i_*(*z*), and are added to the high pass samples. Then, the updated high-pass samples are multiplied by the time domain equivalent of *t_i_*(*z*) and are added to the low-pass samples. If a diagonal matrix is present in the factorization, the low pass coefficients are multiplied by *K* and the high-pass coefficients are multiplied by *K*^−1^. The polyphase-based wavelet transform in lifting scheme is shown in Figure 2.

In this paper, the wavelet “db4” is utilized to construct the wavelet method. The coefficients of the filter are shown in Table 1.

**Figure 3.**
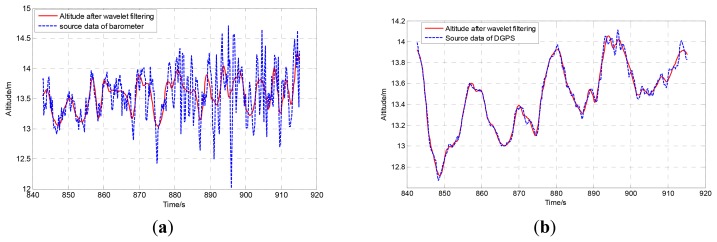
(**a**) The comparison between the original barometer data and the wavelet filtered data. (**b**) The comparison between the original DGPS data and the wavelet filtered data.

The comparisons of original data and the wavelet filtered data of barometer and DGPS are shown in Figure 3. Obviously, the wavelet method can filter out the high frequency noise effectively.

## 4. The Adaptive Extended Kalman Filter

Since the measurement noise structure has changed greatly after wavelet filtering, experiential value or the statistics of partial noise cannot be used to provide a good description of measurement noise covariance, therefore, an AEKF is proposed to estimate the measurement noise covariance in real time to improve altitude information.

Since the nonlinear dynamic equation of SUAR is continuous and the measurements are a discrete series, a continuous-discrete EKF is proposed to fuse altitude sensor information. In EKF, the state equation and measurement equation can be expressed as:

(15) $$\{\begin{array}{l} \dot{X}(t)=f(X(t),t)+B(t)u(t)+W(t)\\ Z_{k}=H_{k}X_{k}+V_{k} \end{array}$$

where, *k* is the number of time step. *X*(*t*) and *Z_k_* are the state vector and measurement vector respectively. *f*(*X*(*t*), *t*) is nonlinear ordinary differential equations, and *H_k_* is the measurement matrix. *B*(*t*) is the input matrix, *u*(*t*) is the input vector, *W*(*t*), *V_k_* are the system noise and measurement noise vector respectively. Besides, the system noise and the measurement noise are uncorrelated, and the system noise can be treated as Gaussian white noise.

In EKF, measurement noise covariance matrix *R* plays an important role in obtaining a converged filter result. If the value of *R* is small, unreliable results will be obtained, and a big value of the diagonal elements of *R* can lead to filter divergence. In traditional EKF, the measurement noise is treated as Gaussian white noise, however, the measurement noise structure has changed greatly after wavelet filtering. Using traditional experiment values or partial statistics of sampling data as measurement noise matrix, the filtering speed will become slow, and filtering performance will become bad, therefore, the sub-optimal and unbiased maximum *a posteriori* method is proposed to estimate *R* in real time. As shown in Equation (21), the current *R* is updated by the innovation *ε_k_* and *R* at previous time. The filter consists of the following stages:

1. The prediction stage:
  (16) $${\hat{X}}_{k/k-1}={\hat{X}}_{k-1}+{\left\{f\left[{\hat{X}}_{k-1},t_{k-1}\right]+B(t_{k-1})u(t_{k-1})\right\}}^{T}$$
  (17) $$P_{k/k-1}=\emptyset_{k,k-1}P_{k-1}\emptyset_{k,k-1}^{T}+Q_{k-1}$$
  (18) $${\hat{Z}}_{k/k-1}=H_{k}{\hat{X}}_{k-1}$$
2. The update stage:
  (19) $${ɛ}_{k}=Z_{k}-{\hat{Z}}_{k/k-1}$$
  (20) $${\hat{X}}_{k}={\hat{X}}_{k/k-1}+K_{k-1}{ɛ}_{k-1}$$
  (21) $$R_{k}=\left(1-\frac{1}{k}\right)R_{k-1}+\frac{1}{k}({ɛ}_{k}{ɛ}_{k}^{T}-H_{k}P_{k}H_{k}^{T})$$
  (22) $$K_{k}=P_{k/k-1}H_{k}^{T}\;[H_{k}P_{k/k-1}H_{k}^{T}+R_{k}]$$
  (23) $$P_{k}=(I-K_{k}H_{k})P_{k/k-1}{\left(I-K_{k}H_{k}\right)}^{T}+K_{k}R_{k}K_{k}^{T}$$
  where *X̂_k_*_/_*_k_*_−1_ is the predicted measurement vector for the next epoch, *Ẑ_k_*_/_*_k_*_−1_ and *P_k_*_/_*_k_*_−1_ are the predicted measurement vector and the predicted state covariance matrix respectively. Ø*_k,k_*_−1_ is the transition matrix after discretization. The innovation *ε_k_* is the difference between the real observations and its estimated values. *T* is the sampling time. *Q_k_* is the system noise covariance matrix. *K_k_* is the gain matrix, *P_k_* is the estimated state covariance matrix, and *R_k_* is the covariance matrix of measurement noise based on the maximum a posteriori adaptive method. Using *ε_k_*, $H_{k}P_{k/k-1}H_{k}^{T}$ and initial experiment value *R*_0_, the measurement noise covariance matrix can be estimated in real time to improve filtering performance.

## 5. Experiment

### 5.1. Hardware System

**Figure 4.**
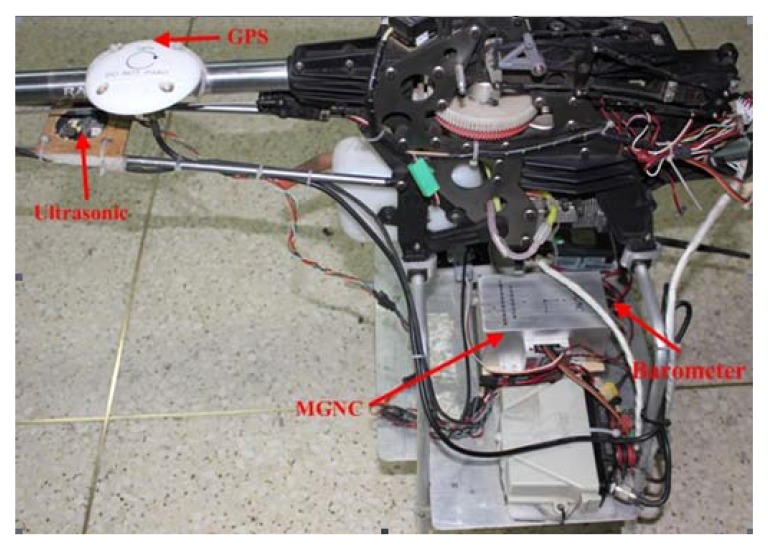
The Raptor-90 helicopter.

Experiments were conducted on a radio-controlled Raptor 90 helicopter, shown in Figure 4. The SUAR is 1.3 m length and 1.46 m span. The total weight is 5 kg, including two liter fuels, a light weight DGPS receiver, and radio telemeter system. The SUAR is powered by a piston engine running on a mixture of methanol and oil. Five servos are used to control the tail, the longitudinal cyclic input, the lateral cyclic input, the collective and the throttle. The longitudinal vertical direction can be stabilized by using the collective and pitch cyclic. Meanwhile, the lateral direction can be controlled by using the roll-cyclic and collective. The heading can be controlled by the tail.

For SUAR, there exist weight and size constraints for onboard control components. Thus, a micro guidance navigation control (MGNC) system with little weight was self-developed to realize stable control. The MGNC is only 207 g in weight, with a size of 120 mm × 61 mm × 48 mm. It consists of a horizontal main board, housing three angular rate sensors, two 2-axis accelerometers and a barometer. The barometer, DGPS, and ultrasonic sensor are used to provide altitude information for the SUAR system. The MPXA6115 barometer, produced by Freescale Semiconductor Company, has a range of 15 kPa∼115 kPa. The DGPS module employs the Novatel RTK, whose position accuracy is about 0.02 m, and the range of the Mini-S electrostatic ultrasonic transducer is from 0.15 m to 6.05 m.

### 5.2. Static Distance Test

To test the effectiveness of the proposed information fusion method, a static distance test has been done on the stairs. A six-floor building is chosen as the basis for its high precision measurement. Three marking points are selected on the stairs. The distances between points and ground have been tested by flexible rulers and the distances are 5.12 m (first point), 9.32 m (second point) and 13.52 m (third point). The SUAR is stretched to measure the distance between the current point and the ground. Besides, the sampling time is 60 s per point.

**Figure 5.**
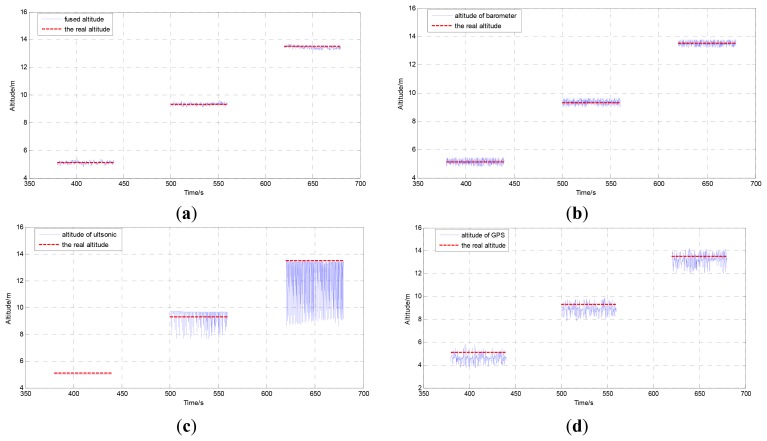
(**a**) The comparison between the output of AEKF and the real altitude. (**b**) The comparison between the output of barometer and the real altitude. (**c**) The comparison between the output of ultrasonic and the real altitude. (**d**) The comparison between the output of DGPS and the real altitude.

The comparison result of the proposed method and the real altitude, the output of the barometer and the real altitude, the output of the DGPS and the real altitude, the output of the ultrasonic sensor and the real altitude are shown in Figure 5. The measurement results of each sensor are shown in Table 2. It is easy to see that the proposed AEKF method has the best performance. Since there exist barriers for GPS in the building, the maximum error reaches to 1.49 m. Without airflow disturbance, the barometer can provide good measurement results, and the standard deviation is 26 percent of the DGPS after initial alignment. The ultrasonic sensor can provide high performance altitude information under 6 m. The mean error at first point is below 0.11 m. When altitude surpasses the 6 m, the performance of ultrasonic is decreased greatly.

### 5.3. Hovering Flight Test

**Figure 6.**
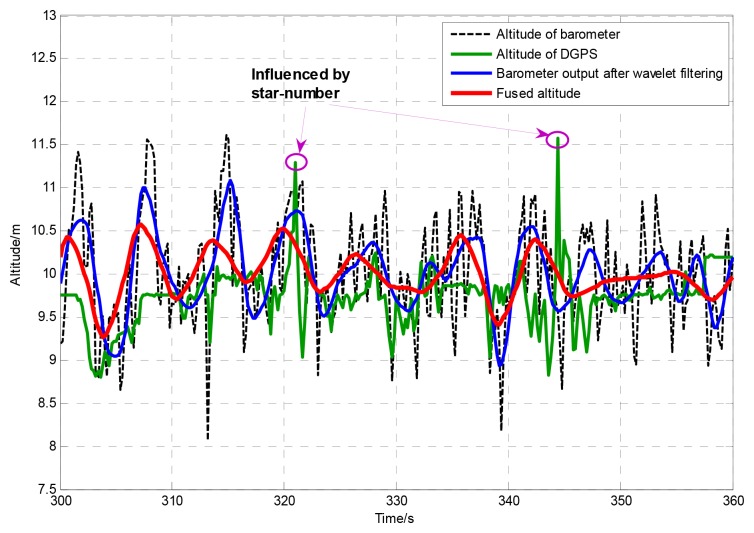
The altitude generated by the AEKF method, barometer and DGPS in a hovering process.

To test the dynamic performance of the proposed method, a hovering flight test has been done on the SUAR system. Under a 3.4 m/s wind disturbance, the SUAR hovers in the air at 10 m altitude. The LQR control method has been used to adjust altitude and position in real time [22]. Since the planned altitude surpasses the upper limit of ultrasonic sensor, DGPS and barometer are used to provide altitude information for the SUAR system. The altitude generated by the AEKF method, barometer and DGPS are shown in Figure 6. The mean error of the adaptive EKF is only 0.214 m, and the standard deviation is 0.169 m. With the proposed AEKF, the SAUR can realize stable hovering control. Furthermore, it is easy to see that the altitude measured by the barometer fluctuates greatly. Since there exists wind disturbance, the fluctuation of barometer surpasses 1 m. Without shelter, the output of DGPS can provide high performance measurement in short periods. With the fluctuation of the star number, the DGPS output is not so reliable.

### 5.4. Autonomous Landing

**Figure 7.**
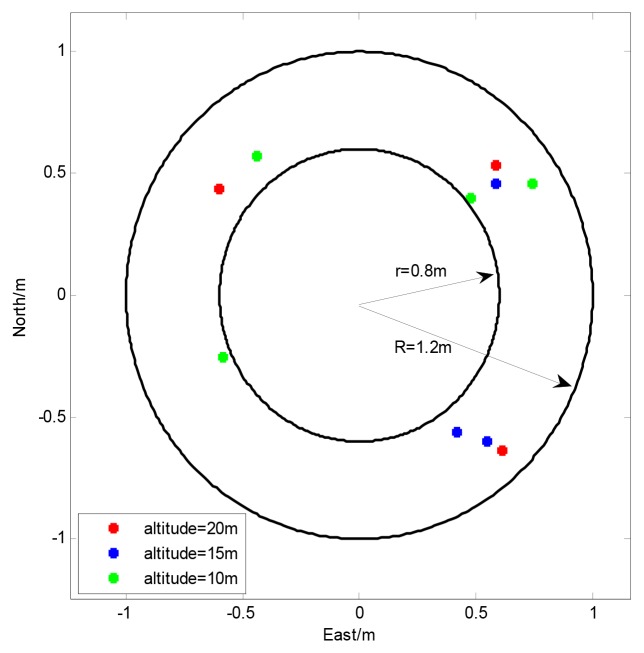
The result of autonomous landing tests from different altitudes.

To test the effectiveness of the proposed method, a series of autonomous landing tests have been done on the SUAR system with the adaptive radial basis function neural network and pilot model. When the SUAR received an autonomous landing command, it changed work station, and flew to the planned hovering point (0,0,10). To satisfy the criteria for position error, speed error and heading error, the SUAR hovered at the planned hovering point. With the constant adjustment for the planned hovering altitude, SAUR descends with hovering stations. Finally, the SUAR landed on the ground. Ten landing tests were conducted from different altitudes, while the wind velocity was less than 3 m/s. The landing results are shown in Figure 7. With the proposed adaptive altitude information fusion method, the SUAR can realize stable autonomous landings, and the average Euclidean distance from the landing target is about 0.67 m. Compared with the navigation system with camera [23,24], the SUAR can get achieve similar landing performance.

**Figure 8.**
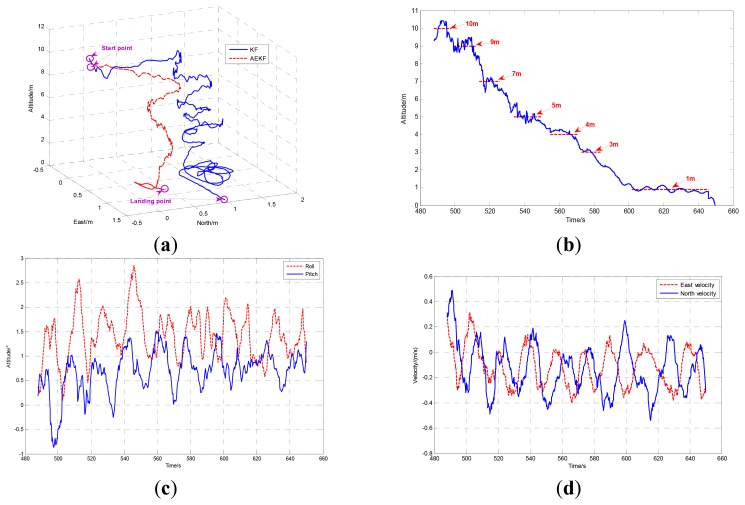
(**a**) The comparison of 3D trajectory of the SUAR with KF and AEKF method in the landing process. (**b**) The altitude trajectory of SUAR in autonomous landing process. (**c**) The pitch and roll angles in autonomous landing process. (**d**) The velocities in two directions.

The comparison of landing performance with AEKF and KF [13] which fuses SINS and DGPS is shown in Figure 8. Using the AEKF, SUAR realized a stable autonomous landing with 0.75 m and 0.45 m error in the East and North directions from the planned landing point. Compared with the KF, the AEFK has much better performance in the autonomous landing process. The altitude, attitude and velocity of the autonomous landing using AEKF are shown in Figure 8(b–d) respectively.

## 6. Conclusions

In this paper, an adaptive information fusion method based on wavelet decomposition and reconstruction is proposed to improve the accuracy and reliability of altitude measurement information in the landing process for a SUAR. With the proposed method, the high frequency noises in sensors can be eliminated greatly, and then high performance altitude information can be fused to provide support for SUAR in the autonomous landing process. The effectiveness of the proposed method has been demonstrated by static tests, hovering tests and a series of autonomous landing tests.

## Figures and Tables

**Table 1.** The coefficients of the “db4” filter.

*h*_0_	**0.48296291314453**	*g*_0_	**0.12940952255126**
*h*_1_	0.83651630373780	*g*_1_	0.22414386804201
*h*_2_	0.22414386804201	*g*_2_	−0.83651630373780
*h*_3_	−0.12940952255126	*g*_3_	0.48296291314453

**Table 2.** Altitude accuracies for AEKF and each sensor (in meters).

	**AEKF**	**Barometer**	**Ultrasonic**	**DGPS**
**Max. absolute error**	0.31	0.32	4.94	1.49
**Mean error**	0.08	0.14	0.44	0.49
**Standard deviation**	0.098	0.16	0.94	0.41
